# Long-Term Stability of the NIST Standard Ultrasonic Source

**DOI:** 10.6028/jres.113.021

**Published:** 2008-10-01

**Authors:** Steven E. Fick

**Affiliations:** National Institute of Standards and Technology, Gaithersburg, MD 20899

**Keywords:** long term stability, radiation force balance, standard ultrasonic source, ultrasonic power

## Abstract

The National Institute of Standards and Technology (NIST) Standard Ultrasonic Source (SUS) is a system comprising a transducer capable of output power levels up to 1 W at multiple frequencies between 1 MHz and 30 MHz, and an electrical impedance-matching network that allows the system to be driven by a conventional 50 Ω rf (radio-frequency) source. It is designed to allow interlaboratory replication of ultrasonic power levels with high accuracy using inexpensive readily available ancillary equipment.

The SUS was offered for sale for 14 years (1985 to 1999). Each system was furnished with data for the set of calibration points (combinations of power level and frequency) specified by the customer. Of the systems that had been ordered with some calibration points in common, three were returned more than once to NIST for recalibration. Another system retained at NIST has been recalibrated periodically since 1984. The collective data for these systems comprise 9 calibration points and 102 measurements spanning a 17 year interval ending in 2001, the last year NIST ultrasonic power measurement services were available to the public.

These data have been analyzed to compare variations in output power with frequency, power level, and time elapsed since the first calibration. The results verify the claim, made in the instruction sheet furnished with every SUS, that “long-term drift, if any, in the calibration of NIST Standard Sources is insignificant compared to the uncertainties associated with a single measurement of ultrasonic power by any method available at NIST.”

## 1. Introduction

Research addressing the measurement and dissemination of ultrasonic power levels began at the National Bureau of Standards (NBS) in the 1970s, in response to the increasing needs of the medical ultrasonics community. The initial outcomes of this research included the design and construction of two instruments—a calorimeter [1] and a radiation force balance (RFB) [2–5]—and the establishment, in 1977, of an ultrasonic power measurement service.

The most frequently requested measurement service was the determination of the effective radiation conductance, *G_r_*, of transducers intended to be used as power transfer standards. Defined by

$$G_{r}=P/V^{2}$$ (1)

where *P* is the output power level in watts and *V* is the root-mean-square (rms) radio-frequency (rf) voltage applied to the transducer, this parameter can be used by the customer to generate arbitrary power levels by simply applying accurately known rf voltages.

By 1980, it was evident that, with the equipment available to typical customers, the accuracy of calibration transfer based on values of *G_r_* was critically affected by the systematic errors associated with rf voltage measurements. Because their missions did not support a standards-lab-grade approach to rf voltage measurements, these customers were fundamentally constrained by the use of radiation conductance.

Accordingly, a scheme was developed at NBS to circumvent the problems of rf voltage measurement by incorporating an rf voltage sensor into the transducer itself. In this scheme, measurements of the applied rf voltage are replaced by measurements of the direct current (dc) output of the sensor for each calibration point specified by the customer. At the customer laboratory, the applied rf voltage level is adjusted to reproduce the dc value supplied by the standards lab for each calibration point. For brevity, this scheme is called the dc-level method (DCLM) in this article.

The SUS was designed to provide NBS customers with an affordable package that could be used to realize the benefits of the DCLM. The SUS transducer incorporates design elements that specifically control every physical effect known or suspected to be capable of inducing changes in performance, both during use and after unlimited long-term storage. Changes in damping are precluded by the use of an air-backed monolithic transducer element directly coupled to the water load, with no intervening layers. The transducer electrodes are applied by vacuum deposition of gold over chrome to maximize the stability of their electrical conductivity and mechanical configuration. Isolation of the transducer element from its surroundings is maximized by its wraparound electrode configuration, its ultrasonically inert guard band comprising 60 % of the transducer area, and by its formed-in-place elastomeric mount made of the same material used to construct absorptive RFB targets. The effects of back loading are minimized by the use of long, thin electrical leads attached to the transducer element with solder joints of minimal mass, and by the large volume of the transducer case. A single-point internal ground for all electrical connections maximizes the electrical shielding provided by the stainless steel transducer case. The other features which distinguish the SUS design from all other known ultrasonic power transfer standard designs have been described extensively elsewhere [6–7].

This article presents the previously unpublished computational procedure required to compare replicate SUS calibration data, and presents the results of a statistical analysis of the long-term stability of the SUS after years of use in the real world.

## 2. SUS Data Comparison

Each element of SUS calibration data consists of a dc value for each calibration point, or combination of power level and operating frequency, requested by the customer. Depending on the dc measuring equipment available to the particular customer, the dc value is expressed either as the voltage measured by a voltmeter of specified input resistance, or as the current applied to a specified load resistance. The dc value considered in this article will be the current applied to a 10 MΩ resistor.

Calibration data are generated at NIST by adjusting the applied rf voltage level until the desired ultrasonic output power level is indicated by the RFB, and then measuring the dc output provided by the SUS. Although an elaborate procedure [3] is used to reduce the measurement uncertainty, for the purposes of this article it suffices to say that the end result is a value of dc output current *I* corresponding to the applied rf voltage level *V* associated with the desired level of ultrasonic output power *P*. When the SUS is recalibrated at some later time *t*, the process is repeated, and the result is a new value of dc current *I_t_*, corresponding to the new value of applied rf voltage *V_t_*, which the customer will use to generate *P* until the next recalibration.

To provide a common basis for stability assessment, let the power level *P_t_* be defined as the value of transducer output power that would be measured at time *t* if the applied rf voltage level were set to the original level *V*. Because any drift in SUS characteristics during the few minutes of time required to make a set of power measurements will be taken into account by the statistics of the data set itself, and keeping in mind that *G_r_* is well known to be independent of the applied rf voltage level, it follows from Eq. (1) that $P/V_{t}^{2}=P_{t}/V^{2}$, and therefore that

$$P_{t}=P{\left(V/V_{t}\right)}^{2}.$$ (2)

**Fig. 1:**
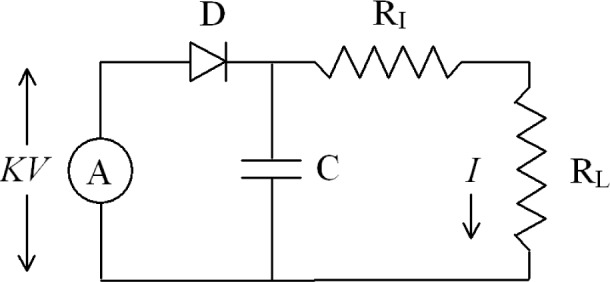
Equivalent circuit of the SUS rf voltage sensor.

Expressing (*V*/*V_t_*)^2^ in terms of *I* and *I_t_* is facilitated by considering the SUS rf voltage sensor in terms of the equivalent circuit shown in Fig. 1.

The five elements of this circuit are:

1. RF voltage source A with output *KV*
2. Rectifier diode D
3. Filter capacitor C
4. Isolating resistor R_I_, and
5. Load resistor R_L_.

The rf voltage source A represents the SUS internal rf voltage attenuator, which reduces the applied rf voltage to accommodate the peak inverse voltage limit of rectifier diode D. Scale factor *K* is defined to be the result of dividing the rms rf voltage at the attenuator input by the peak rf voltage at the attenuator output. Filter capacitor C is charged by pulses of current from D and establishes the dc voltage which induces dc current *I* into the series combination of R_I_ and R_L_. Isolating resistor R_I_ is the equivalent of the resistors used in the real circuit to superimpose the dc current and the applied rf voltage inside the transducer, and to separate the dc current from the applied rf voltage inside the matching network. Load resistor R_L_ is the equivalent of the input resistance of the customer’s dc meter, and any trimming resistors required to establish the specified SUS load resistance.

Because the RC time constants have been chosen to allow the dc voltage across C to approach the peak rf output voltage of A, the circuit can be described by a simple equation:

$$KV=V_{D}+I\left(R_{I}+R_{L}\right)$$ (3)

where *V_D_* is the dc voltage drop across rectifier diode D, and *R_I_* and *R_L_* are the resistances of the resistors.

For the 140 nA to 290 nA range of values of *I* applicable to the data considered in this article, *V_D_* is a logarithmic function of *I* given by

$$V_{D}=a+b\ln I$$ (4)

where parameters *a* and *b* are determined empirically from the results of 120 tests in which the dc voltage drop was measured as a function of 47 levels of dc current between 50 nA and 290 nA applied to 5 sample diodes from the hand-selected batch of diodes used in the construction of all SUS transducers. The values used in the calculations for this article are: *a* = 0.0478013 V, and *b* = −0.0579971 V.

By combining Eqs. (2) through (4), the formula for *P_t_* is found to be:

$$P_{t}=P{\left[\frac{a+b\ln I+I\left(R_{I}+R_{L}\right)}{a+b\ln I_{t}+I_{t}\left(R_{I}+R_{L}\right)}\right]}^{2}.$$ (5)

The error in *P* associated with the empirical determination of *V_D_* varies from 0.02 % to 0.06 % for the data analyzed herein, and is insignificant compared to the various other uncertainty components [3–5] applicable to measurements of *P*.

## 3. Statistical Analysis

A database maintained at NIST contains the results of all SUS calibrations. These results include, for each measured value of *P*, a value of expanded uncertainty [8] obtained using two as the coverage factor by which the corresponding combined relative standard uncertainty *U* was multiplied.

In the interests of both confidentiality and statistical robustness, the data analyzed in this article were selected to meet two criteria:

1. Each calibration point was requested for at least two SUS systems.
2. For each SUS system, the data set for a particular calibration point contains at least three values of *P*, the results of the original calibration and two recalibrations.

These selection criteria are met by 9 calibration points with 102 measurements for 4 SUS systems. For these data, the minimum number of data points per calibration point is 6, the maximum is 30, and the average is 11. The values of *U* for these data vary from 0.007 to 0.015, with average value 0.009.

The data analyzed in this article represent 2 power levels and 7 nominal operating frequencies determined by rounding each SUS operating frequency to the nearest megahertz, to allow the appropriate comparisons of SUS data for the same harmonic order [6].

For each calibration point and SUS, all possible values of *P_t_* were calculated using Eq. (5). Next, the average of *P* and all values of *P_t_* was computed. Multiplication by the reciprocal of this average was then used to convert each value of *P_t_* to a normalized output power level *P_n_*. Because this procedure causes the average of all values of *P_n_* to be unity, the deviation for each value of *P_n_* is given by *D_n_* = *P_n_*−1. To allow exploration of the possibility of bias due to the variations in *U*, the parameter defined by *D_U_* = *D_n_*/*U*, where the subscript *U* denotes normalization with respect to *U*, was computed for all values of *D_n_*.

The deviations *D_U_* and the normalized output power levels *P_n_* for every calibration point and SUS were aggregated and sorted to explore dependencies on frequency, power level, and time elapsed since the first calibration. The analyses described next were applied independently to both parameters. The end results differed indistinguishably. Because of their relative ease of interpretation, only the results for *P_n_* are presented.

### 3.1 Probability Distribution

The aggregated data represent the combined effects of errors in the measurements of *P* and possible instability of the SUS systems. Errors in the measurement of *P* are expected to be normally distributed because the RFB design and operating procedures take into account all known systematic effects[3–5]. The statistical possibilities for SUS instability are more difficult to predict, however, because at least one possible cause, drift in the values of the voltage divider capacitors for a particular transducer, is more often monotonic than random.

**Fig. 2:**
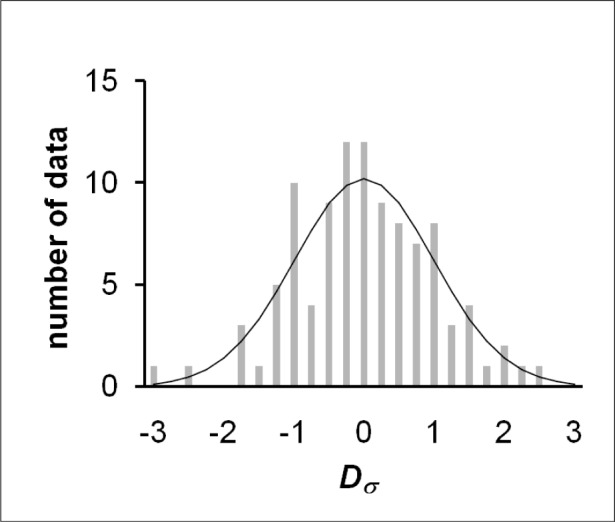
Histogram of normalized deviations.

Investigation of the underlying probability distribution of the aggregated data is facilitated by computing for each value of *D_n_* a new parameter defined by *D_σ_* = *D_n_*/*σ*, where *σ* is the standard deviation of all 102 values of *P_n_*. A histogram of all values of *D_σ_* is shown in Fig. 2, which also shows a least-squares fit normal distribution function. The general appearance of the histogram—|*D_σ_* | < 3 for all data, |*D_σ_* | < 2 for 94 % of the data, and |*D_σ_* | < 1 for 60 % of the data—supports the hypothesis that the underlying distribution is normal. This hypothesis was explored further by applying the Anderson-Darling test [9] to the aggregated *P_n_* data. The value of the adjusted Anderson-Darling test statistic [10] is 0.071, well less than the critical value 0.787 for which the hypothesis would be rejected at the 95 % confidence level. For purposes of further analysis in this article, the data will be considered to be normally distributed.

### 3.2 Variation With Frequency

**Fig. 3:**
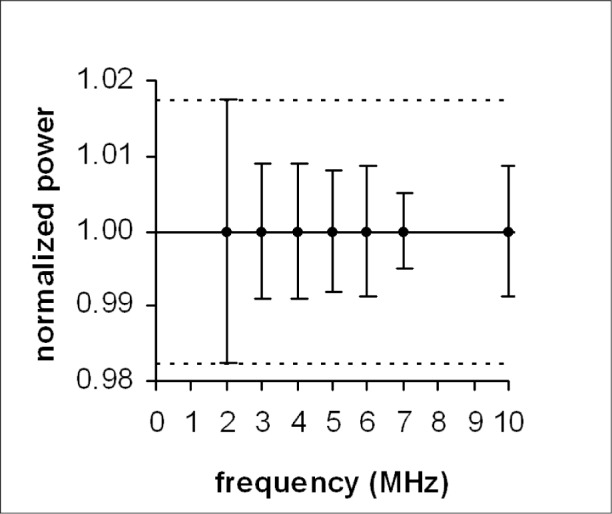
Variation of normalized power (*P_n_*) with frequency.

Analysis of the frequency dependence was done by sorting the aggregated *P_n_* data into groups, one for each of the 7 different nominal frequencies. Each group contains the data for all calibration points corresponding to its particular frequency. The mean of all values of *P_n_* in each group is unity, because the procedure for computing the values of *P_n_* for each calibration point causes the average of all values of *P_n_* to be unity for each calibration point. Variation with frequency is indicated by the standard deviations of the data in each group. Figure 3 shows the mean values as data, and the standard deviations as error bars. For clarity, and to provide a conservative comparison of the standard deviations of *P_n_* with the associated uncertainties, the dashed lines show the limits set by the lowest of the average expanded uncertainties computed for each of the 7 frequencies.

As expected, for each frequency the variations indicated by the standard deviations do not exceed the uncertainty limits, and there is no obvious trend.

### 3.3 Variation With Power Level

**Fig. 4:**
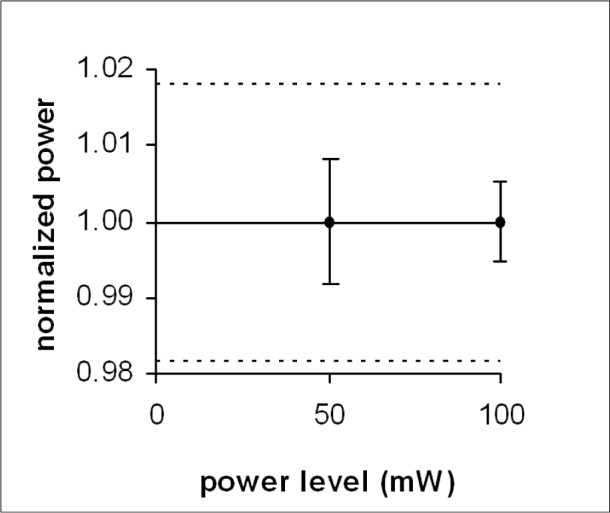
Variation of normalized power (*P_n_*) with power level.

Analysis of the dependence on power level was done by sorting the aggregated *P_n_* data into two groups, one for each of the two different power levels. The mean of all values of *P_n_* in each group is unity, and variation with frequency is indicated by the standard deviations of the data in each group. Figure 4 shows the mean values as data, and the standard deviations as error bars. The dashed lines show the limits set by the lower of the average values of *U* computed for the two power levels.

The standard deviations for the two power levels are well within the uncertainty limits, and sufficiently similar in magnitude that no trend is apparent.

### 3.4 Variation With Time Elapsed Since the First Calibration

Analysis of the dependence on elapsed time was begun by sorting the aggregated *P_n_* data by time elapsed since the first calibration. In the interests of clarity, the data were then combined into 5 groups chosen to include data from at least two SUS systems. Because none of these groups includes all of the data for a particular calibration point, the average value of *P_n_* for each group will not necessarily be unity. The average values of elapsed time, and the average values and standard deviations of *P_n_* were calculated for each group.

**Fig. 5:**
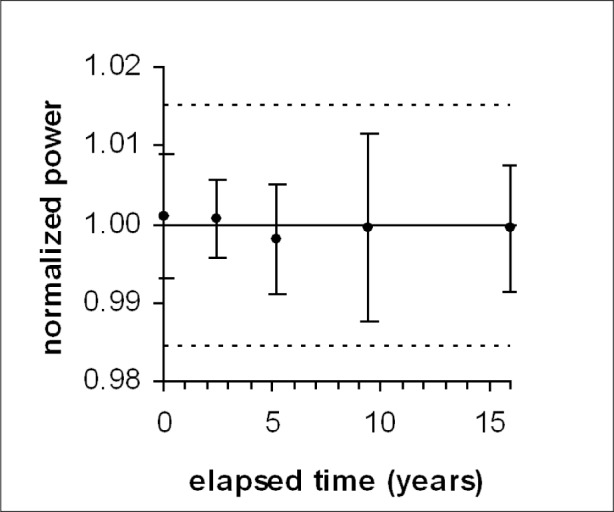
Variation of normalized power (*P_n_*) with elapsed time.

Average values of *P_n_* are plotted in Fig. 5, in which the error bars show the standard deviations. The dashed lines show the limits set by the lowest of the average values of *U* computed for each of the 5 average values of elapsed time. Although significant variations in average and standard deviation are evident, no trend is apparent.

The significance of drift to the use of the SUS as a transfer standard justifies further analysis by means beyond visual inspection.

Least-squares linear regression analysis [11] was applied to the aggregated *P_n_* data and the associated 102 values of elapsed time in years. The range of the estimated slope is −0.000291 to 0.000336, stated at the 95 % (2 standard error) confidence level used for all linear regression results in this article. The fact that its range includes zero is generally considered to indicate that an estimated slope is statistically indistinguishable from zero [12]. In practical terms, the drift rate of the SUS power measurements considered in this article can also be considered to be indistinguishable from zero.

The aggregated *P_n_* data represent 4 SUS systems, 9 calibration points, 30 different test dates spanning more than 17 years, and 102 measurements. The diversity of factors applicable to these data is considered to be sufficient to make the SUS design one of two predominant common factors to which the statistical characteristics of the data are attributable. Because the other common factor is the single RFB with which all measurements were made, the analytical results can also be taken to confirm that all known RFB drift mechanisms have been taken into account adequately in RFB operating and data processing procedures.

### 3.5 Performance of Individual SUS Systems

Least-squares linear regression analysis against elapsed time was also done for the *P_n_* data from each of the 4 SUS systems, designated A, B, C, and D for clarity. The estimated drift rates for systems A and B are indistinguishable from zero. For systems C and D, the magnitudes of the estimated drift rates are nonzero and nearly the same, with average value 0.069 ± 0.053 percent per year. However, the drift rate is positive for system C and negative for system D. These results are fully consistent with the results for the aggregated data, and are presented only to verify the absence of computational errors in the regression analysis.

The SUS drift rates reported in this article should not be used as a basis for comparison with SUS drift rates determined by other means, because these SUS drift rates typically are determined primarily by the performance of the ultrasonic power measurement equipment, rather than the performance of the SUS. Such comparisons could be meaningful only if the measurements spanned many years and were made by one of the few national metrology institutes in the world [13] capable of the measurement uncertainties applicable to the data analyzed herein.

## 4. Conclusion

A computational procedure for comparison of replicate SUS calibration data has been presented.

A data set composed of the results of 102 measurements representing 4 SUS systems, 9 calibration points, 7 frequencies, 2 power levels, and 29 intervals of time has been analyzed.

The drift rate extracted from these data was found to be indistinguishable from zero at the 95 % level of statistical confidence. This result verifies the claim, made in the instruction sheet furnished with every SUS, that “long-term drift, if any, in the calibration of NIST Standard Sources is insignificant compared to the uncertainties associated with a single measurement of ultrasonic power by any method available at NIST.”
